# Prognostic impact of examined lymph node count in pT1N0M0 esophageal cancer: A population‐based study

**DOI:** 10.1111/1759-7714.13130

**Published:** 2019-06-24

**Authors:** Ying Liu, Heli Yang, Hao Fu, Meng Li, Zhen Feng, Zhongmin Peng, Zhen Liang, Hui Wang

**Affiliations:** ^1^ Department of Thoracic Surgery Shandong Provincial Hospital Affiliated to Shandong First Medical University Jinan China; ^2^ Department of Thoracic Surgery I, Key Laboratory of Carcinogenesis and Translational Research (Ministry of Education) Peking University Cancer Hospital and Institute Beijing China

**Keywords:** Esophageal cancer, examined lymph node count, long‐term survival, population‐based study, prognostic factors

## Abstract

**Background:**

Research on the impact of examined lymph node (ELN) count on node‐negative esophageal cancer (EC) especially pT1N0M0 EC is inadequate. This study was designed to analyze the prognostic impact of ELN count on pT1N0M0 EC.

**Methods:**

Data of resected pT1N0M0 EC patients between 1988 and 2015 were extracted from the United States Surveillance, Epidemiology, and End Results (SEER) database. The association between ELN count and overall survival (OS) or esophageal cancer‐specific survival (ECSS) were investigated. Factors that may predict the outcome were identified using the Kaplan‐Meier method and the Cox proportional‐hazards model.

**Results:**

A total of 906 patients who underwent resection with at least one lymph node (LN) retrieved met our criteria. The cumulative 5‐year OS was 67.6%, while the cumulative 5‐year ECSS was 75.4%. X‐Tile analysis showed that 12 was the optimal cutoff value for ELN count in terms of both OS (χ2 = 28.764, *P* < 0.0001) and ECSS (χ2 = 15.668, *P* = 0.0026). A Kaplan‐Meier survival analysis and log‐rank comparison revealed that ELN > 12 was significantly associated with better OS (HR, 0.532; 95% CI, 0.421–0.672; *P* < 0.001) and ECSS (HR, 0.561; 95% CI, 0.420–0.749; *P* < 0.001) rates than ELN ≤12. The OS and ECSS benefit of increasing ELN count were also reflected in the multivariate analysis after adjustment for age, sex, race, marital status, location, T stage, tumor size, pathology, and differentiation.

**Conclusions:**

These findings indicated that greater number of ELN count exhibits prognostic significance in pT1N0M0 EC. We recommend more than 12 LNs should be examined in pT1N0M0 EC.

## Introduction

Esophageal cancer (EC) ranks sixth among the causes of cancer related mortality globally, with approximately 508 585 deaths worldwide in 2018.^1^ In recent years, multi‐modality treatment dominated by surgery has improved the prognosis of patients to a certain degree. However, the overall prognosis remains poor with a 5‐year survival rate estimated at 15% to 25%.^2^ However, with recent advances in endoscopic surveillance and early detection,^3^, ^4^ early‐stage cancers (T1N0M0) are being diagnosed more frequently. In early‐stage EC, lesions are limited to the mucosa (T1a) or submucosa (T1b),^5^ and this diagnosis comprises approximately 20% of all initial diagnoses.^6^ Prognosis for these patients is better and a 5‐year overall survival (OS) rate of 90% has been reported in resected T1a patients.^7^ Endoscopic therapy appears to be comparable with esophagectomy in terms of mid‐ and long‐term EC‐related mortality,^8^ but several risk factors may preclude adequate treatment with endoscopic therapy. Endoscopic therapy could just be therapeutic when a lesion ≤2 cm in diameter is fully removed with clear lateral and deep margins and histopathologic assessment demonstrates well or moderate differentiation, invasion no deeper than the superficial submucosa, and no lymphovascular invasion (LVI).^9^, ^10^ Esophagectomy should continue to remain the standard treatment in patients with T1N0M0 EC.^10^

Surgery may not only resect the lesion and potential precancerous lesions to the maximum extent, but also dissect the potential metastatic lymph nodes (LN) to help accurate staging and improve the prognosis. In the past decade, many studies have examined the impact of examined lymph node (ELN) count on the survival of patients with cancer, and a higher number of ELNs is associated with a better prognosis.^11^, ^12^, ^13^, ^14^ However, few studies have considered the relationship between the ELN count and survival in patients with pT1N0M0 EC. The minimum number of LNs requiring resection and its prognostic effect on the long‐term survival of patients with pT1N0M0 EC remain undetermined. Hence, in this report, we performed a population‐based retrospective analysis of the United States Surveillance, Epidemiology, and End Results (SEER) database to investigate whether the ELN count in resected pT1N0M0 EC patients acts as a prognostic factor for the OS and esophageal cancer‐specific survival (ECSS).

## Methods

### Data source

The data used in this study were extracted from the SEER registry program of the National Cancer Institute. SEER is a population‐based database of the National Cancer Institute on cancer incidence and survival. The population‐based cancer registries included 18 registries and covered approximately 28% of the US population and were analyzed using SEER*‐Stat software. SEER database is freely available with patient anonymization, and approval from the institutional review board is therefore not required.

### Study population

We identified all patients with primary EC localized to the upper, middle, and lower esophagus from 1988 to 2015. The primary site and morphology codes C15.0 and C15.3 were used to identify tumors localized to the upper third of the esophagus, C15.4 was used to identify the middle third of esophagus, and C15.2 and C15.5 were used to identify the lower third of the esophagus. All patients were microscopically confirmed. CS extension (2004+) and EOD 10 extent (1988–2003) were used to define the T stage of the cancer. We defined T1 stage EC as lesions involving the mucosa (T1a) or submucosa (T1b). EC was the only primary cancer and pathologic LN should be negative. Histologic codes 8140, 8144, 8210, 8211, 8255, 8260, 8263, 8310, 8480, and 8481 were used to define adenocarcinomas; codes 8052, 8070, 8071, 8072, 8074, and 8083 for squamous cell carcinomas; and all other remaining codes as other histology. Surgery codes 40, 50 and 60 were used for patients who underwent esophagectomy before 1997. For patients after 1998, surgery codes 30, 40, 50–55, and 80 were used. The surgical treatments included partial esophagectomy, total esophagectomy, esophagectomy with laryngectomy and/or gastrectomy, and esophagectomy NOS. Those patients who received neoadjuvant radiation were kept within the analysis; information on chemotherapy is not provided in the SEER data. Exclusion criteria were as follows: patients under the age of 18 at diagnosis; patients with missing or incomplete information regarding race, marital status, tumor location, differentiation, type of surgery, survival status and cause of death. Patients with follow‐up status of less than one month were excluded from the study.

The following information was obtained for each patient from the SEER database: patient demographics (such as year of diagnosis, sex, age at diagnosis, race, and marital status); clinicopathological characteristics (involving primary site, tumor size, histologic type, differentiation, and T stage) and survival information (such as survival months, vital status and cancer‐specific death).

ELN count was abstracted using SEER codes. ELN count is the total number of regional LNs that were removed and examined by the pathologist. Code 00 is determined as no LNs were examined. Codes 01 to 89 are considered as exact number of LNs examined. Code 90 is considered as 90 or more LNs were examined. Codes 95 to 98 included categories where the number of LNs was unknown or not stated in the pathology report or LNs removed were not documented as part of the surgical procedure were excluded in this study. Code 99 is considered as unknown whether the LNs were examined or not applicable or negative or not stated in the patient record and was also excluded in this study.

The outcomes of this study included OS and ECSS. OS was defined from the date of surgery to the date of death due to any cause. ECSS was defined as the number of months from date of surgery until death due to EC. Patients who died due to other causes or were still alive at the end of the study period were defined as censored. The last follow‐up in this study was on 31 December 2015.

### Statistical analysis

We used the Student's *t*‐test^15^ to compare the differences between continuous variables and χ ^2^ test^16^ for categorical variables. The optimal cutoff values for the ELN count were determined using X‐Tile software (http://www.tissuearray.org/rimmlab), and identified the cutoffs with minimum *P* values from log‐rank χ ^2^ statistics in terms of survival.^17^ The survival rate was calculated using the Kaplan‐Meier method, and a log‐rank test was used to assess the survival differences between groups. A Cox proportional‐hazards model^18^ was used for multivariate analysis. Statistical significance was assumed for a two‐tailed *P*‐value of less than 0.05. All statistical analyses were performed using SPSS (version 22.0, SPSS Inc., Chicago, IL).

## Results

### Patient characteristics and ELN count

**Figure 1:**
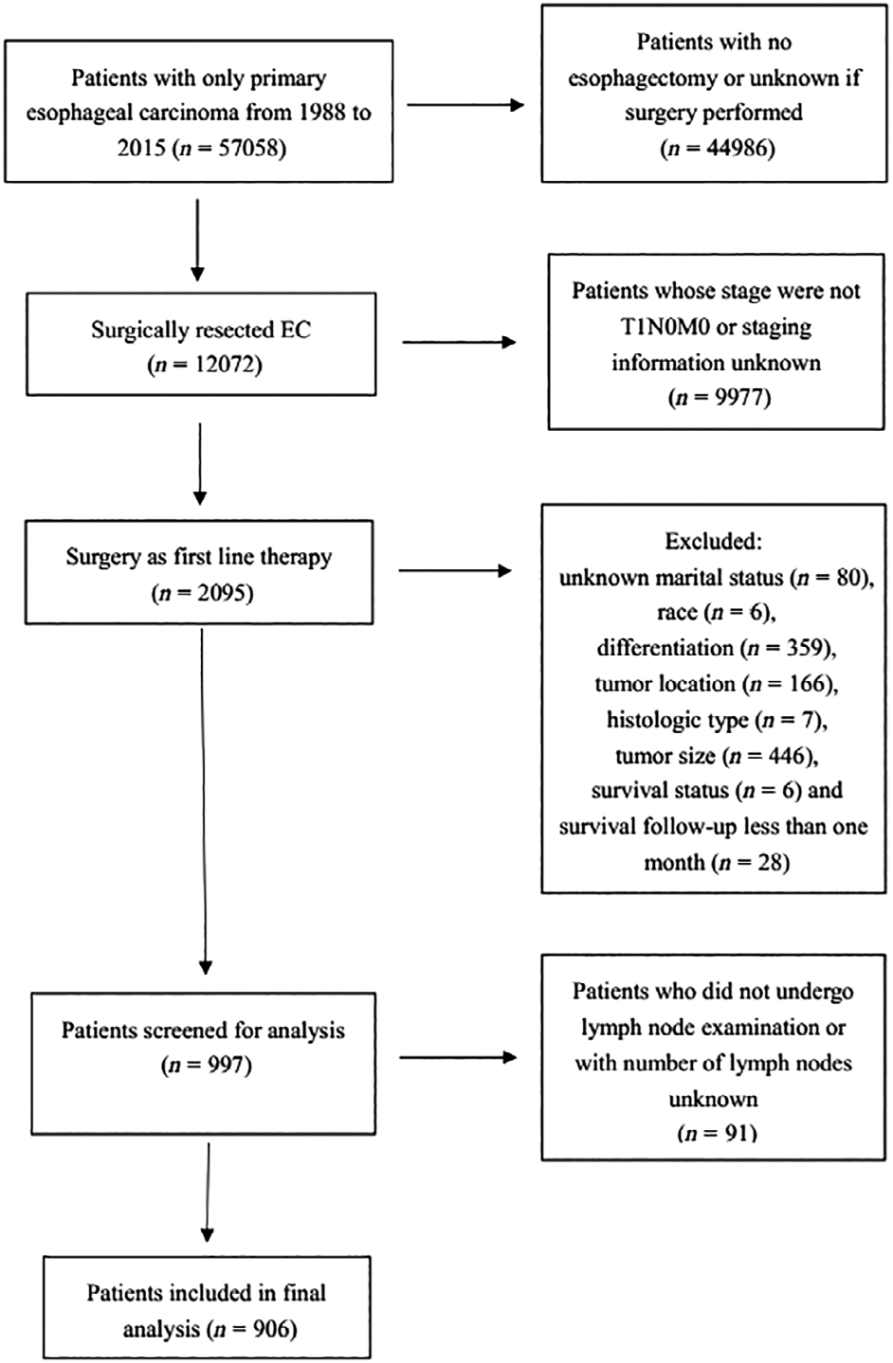
Selection of study cohort.

From 1988 to 2015, a total of 906 patients (mean [±SD] age: 63.8 ± 9.4 years; 82.7% males; 90.7% whites) with microscopically confirmed stage T1a (38.5%) and stage T1b (61.5%) were identified. The flow diagram for patient selection is presented in Fig 1. The largest proportion of tumors were located in the lower third (82.8%), followed by the middle third (14.3%) and upper third (2.9%) of the esophagus. The adenocarcinomas are the most common ECs constituting approximately 82.0% of the cohort. A total of 84 patients received neoadjuvant radiation therapy. The median ELN count was 10 (interquartile range [IQR], 5 to 17). Patient characteristics are summarized in Table 1.

**Table 1:** Clinicopathologic characteristics of the entire cohort of patients (*n* = 906)

Characteristics	No. (%) of patients
Age, mean ± SD	63.8 ± 9.4
Age group, years	
<60	280 (30.9)
60–75	525 (57.9)
>75	101 (11.1)
Sex	
Male	749 (82.7)
Female	157 (17.3)
Race	
White	822 (90.7)
Others	84 (9.3)
Marital status	
Married	648 (71.5)
Single	258 (28.5)
Location	
Upper third	26 (2.9)
Middle third	130 (14.3)
Lower third	750 (82.8)
T stage	
T1a	349 (38.5)
T1b	557 (61.5)
Tumor size	21.4 ± 17.5
Pathology	
AC	743 (82.0)
SQC	134 (14.8)
Others	29 (3.2)
Differentiation	
I	154 (17.0)
II	453 (50.0)
III	279 (30.8)
IV	20 (2.2)
ELN count	
1–5	228 (25.2)
6–10	227 (25.1)
11–17	226 (24.9)
>17	225 (24.8)

AC, adenocarcinoma; SQC, squamous cell carcinoma.

### Survival analysis and identification of the optimal cutoff value for ELN count in pT1N0M0 EC

**Figure 2:**
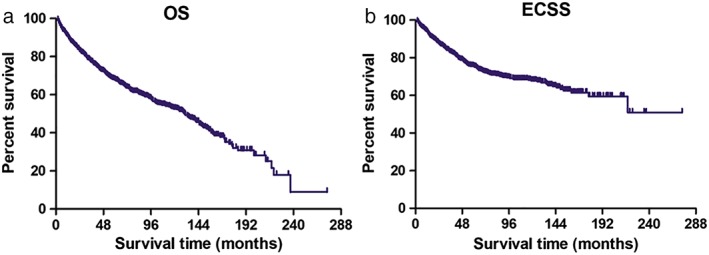
(**a**) Overall survival (OS) and (**b**) esophageal cancer–specific survival (ECSS) in 906 patients with pT1N0M0 EC.

The median follow‐up period after surgical resection was 54 (range, 2–274) months. A total of 376 deaths were reported during the follow‐up period, where 233 deaths were due to EC and 143 deaths due to other causes. The cumulative 5‐year OS was 67.6%, while the cumulative 5‐year ECSS was 75.4% (Fig 2).

**Figure 3:**
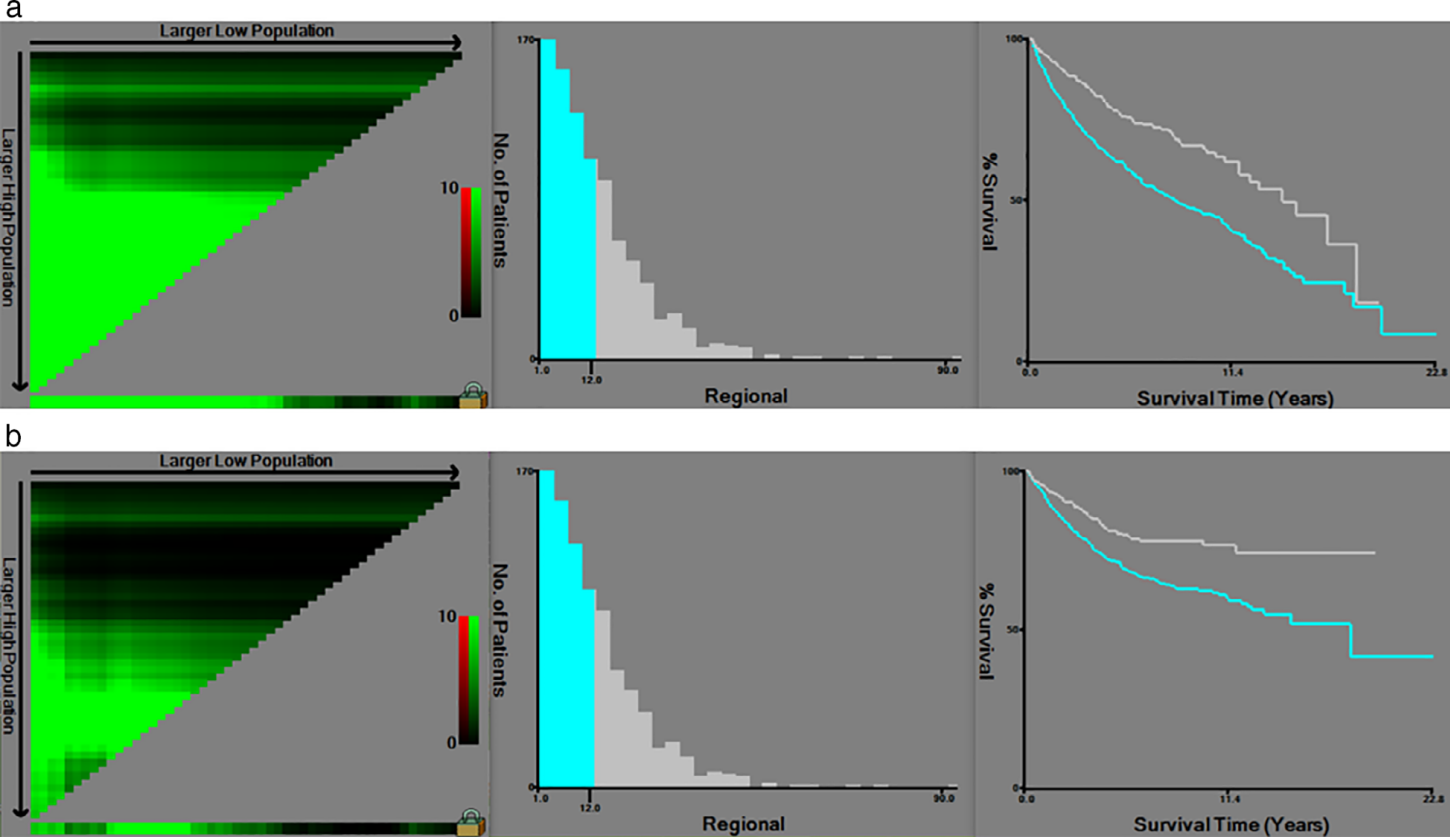
X‐tile analysis of survival data in pT1N0M0 esophageal cancer in terms of both (**a**) overall survival and (**b**) esophageal cancer‐specific survival. X‐tile plots of the training sets are shown in the left panel, with plots of matched validation sets shown in the smaller inset. The optimal cutoff point is shown on a histogram of the entire cohort (middle panels), and a Kaplan‐Meier plot (right panels).

**Figure 4:**
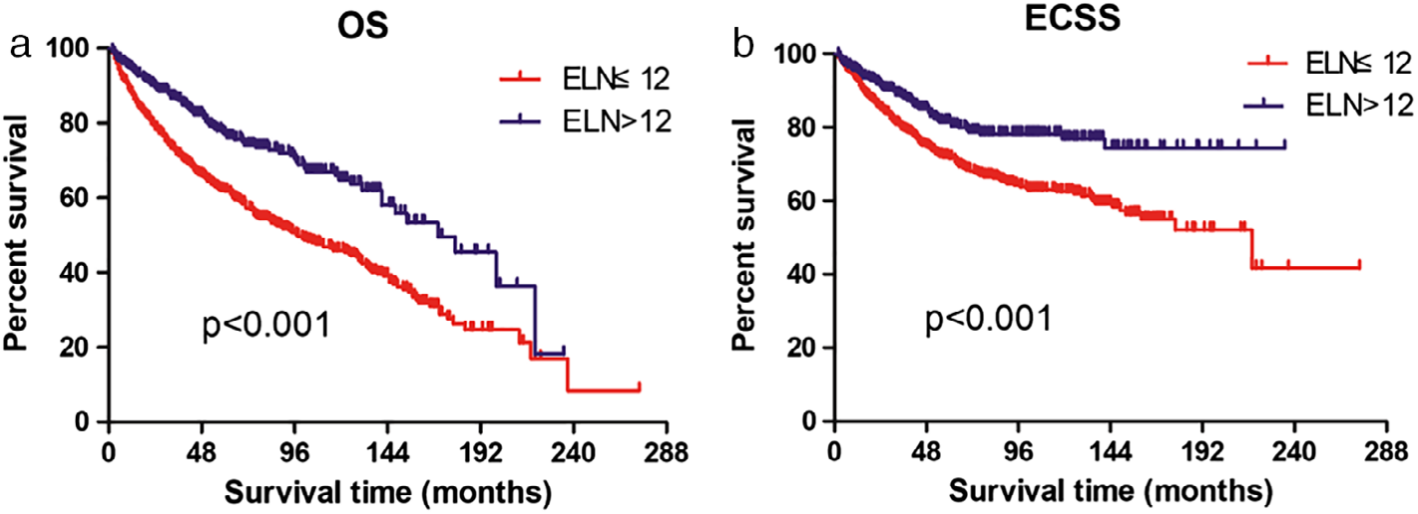
(**a**) Overall survival and (**b**) esophageal cancer–specific survival by ELN count in 906 patients with pT1N0M0 EC.

X‐Tile analysis showed that 12 was the optimal cutoff value for ELN count in patients with pT1N0M0 EC in terms of both OS (χ ^2^ = 28.764, *P* < 0.0001) and ECSS (χ ^2^ = 15.668, *P* = 0.0026), (Fig 3). Table 2 compared the clinicopathological factors between EC patients in the ELN ≤12 and ELN > 12 groups, which implied they were comparable based on the possible confounding variables. A Kaplan‐Meier survival analysis and log‐rank comparison revealed that ELN > 12 was significantly associated with better OS (HR, 0.532; 95% CI, 0.421–0.672; *P* < 0.001) and ECSS (HR, 0.561; 95% CI, 0.420–0.749; *P* < 0.001) rates than ELN ≤12 (Fig 4).

**Table 2:** Distribution of clinicopathologic characteristics for the two groups by ELN count

	ELN count (%)		*P*
Variables	ELN count ≤ 12 (*n* = 533)	ELN count > 12 (*n* = 373)	
Age, mean ± SD	64.2 ± 9.6	63.1 ± 8.9	0.101
Age group, years			
<60	156 (29.3)	124 (33.2)	0.290
60–75	312 (58.5)	213 (57.1)	
>75	65 (12.2)	36 (9.7)	
Sex			
Male	446 (83.7)	303 (81.2)	0.339
Female	87 (16.3)	70 (18.8)	
Race			
White	491 (92.1)	331 (88.7)	0.084
Others	42 (7.9)	42 (11.3)	
Marital status			
Married	378 (70.9)	270 (72.4)	0.630
Single	155 (29.1)	103 (27.6)	
Location			
Upper third	19 (3.6)	7 (1.9)	0.133
Middle third	69 (12.9)	61 (16.4)	
Lower third	445 (83.5)	305 (81.8)	
T stage			
T1a	196 (36.8)	153 (41.0)	0.196
T1b	337 (63.2)	220 (59.0)	
Tumor size	21.8 ± 19.0	20.9 ± 14.9	0.399
Pathology			
AC	443 (83.1)	300 (80.4)	0.412
SQC	76 (14.3)	58 (15.5)	
Others	14 (2.6)	15 (4.0)	
Differentiation			
I	83 (15.6)	71 (19.0)	0.400
II	269 (50.5)	184 (49.3)	
III	171 (32.1)	108 (29.0)	
IV	10 (1.9)	10 (2.7)	

AC, adenocarcinoma; SQC, squamous cell carcinoma.

The OS and ECSS benefit of increasing ELN count were also reflected in the multivariate analysis after adjustment for age, sex, race, marital status, location, T stage, tumor size, pathology, and differentiation (Tables 3, 4). Other independent favorable prognostic factors identified both in the OS and ECSS multivariate analysis included younger age at diagnosis, tumor size ≤18 mm and well‐differentiated histology. T stage was also an independent prognostic factor in the ECSS multivariate analysis.

**Table 3:** Cox regression analysis for estimating the risk factors of OS

	Univariate			Multivariate		
Variables	HR	95%CI	*P*	HR	95%CI	*P*
Age group, years			<0.001			<0.001
<60	Ref			Ref		
60–75	1.548	1.214–1.974	<0.001	1.530	1.197–1.956	0.001
>75	2.814	2.025–3.913	<0.001	2.569	1.843–3.582	<0.001
Sex			0.202			NS
Male	Ref			NS		
Female	1.185	0.913–1.536	0.202	NS	NS	NS
Race			0.279			NS
White	Ref			NS		
Others	0.822	0.576–1.172	0.279	NS	NS	NS
Marital status			0.074			NS
Married	Ref			NS		
Single	1.224	0.980–1.528	0.074	NS	NS	NS
Location			0.004			0.090
Upper third	Ref			Ref		
Middle third	1.136	0.661–1.954	0.644	1.331	0.742–2.385	0.337
Lower third	1.539	1.191–1.990	0.001	0.952	0.541–1.676	0.864
T stage			0.001			0.067
T1a	Ref			Ref		
T1b	1.435	1.154–1.783	0.001	1.234	0.985–1.546	0.067
Tumor size			0.001			0.011
≤18	Ref			Ref		
>18	1.400	1.143–1.715	0.001	1.313	1.065–1.619	0.011
Pathology			0.01			0.524
AC	Ref			Ref		
SQC	1.447	1.120–1.869	0.005	1.117	0.821–1.519	0.482
Others	0.781	0.426–1.432	0.425	0.778	0.417–1.453	0.431
Differentiation			0.005			0.044
I	Ref			Ref		
II	1.036	0.761–1.409	0.823	0.880	0.644–1.201	0.420
III	1.501	1.093–2.061	0.012	1.211	0.874–1.677	0.250
IV	0.953	0.467–1.945	0.894	0.786	0.381–1.622	0.515
ELN count			<0.001			<0.001
≤12	Ref			Ref		
>12	0.532	0.421–0.672	<0.001	0.539	0.425–0.683	<0.001

AC, adenocarcinoma; SQC, squamous cell carcinoma; Ref, reference; NS: *P* > 0.05 on univariate analysis.

**Table 4:** Cox regression analysis for estimating the risk factors of ECSS

	Univariate			Multivariate		
Variables	HR	95%CI	*P*	HR	95%CI	*P*
Age group, years			0.006			0.041
<60	Ref			Ref		
60–75	1.383	1.023–1.869	0.035	1.324	0.977–1.795	0.070
>75	2.004	1.298–3.094	0.002	1.719	1.110–2.664	0.015
Sex			0.124			NS
Male	Ref			NS		
Female	1.289	0.933–1.780	0.124	NS	NS	NS
Race			0.899			NS
White	Ref			NS		
Others	1.028	0.673–1.570	0.899	NS	NS	NS
Marital status			0.390			NS
Married	Ref			NS		
Single	1.132	0.853–1.502	0.390	NS	NS	NS
Location			0.009			0.454
Upper third	Ref			Ref		
Middle third	1.107	0.559–2.193	0.770	1.044	0.522–2.089	0.903
Lower third	0.681	0.360–1.290	0.239	0.820	0.418–1.607	0.563
T stage			<0.001			0.018
T1a	Ref			Ref		
T1b	1.811	1.356–2.419	<0.001	1.434	1.065–1.931	0.018
Tumor size			<0.001			0.009
≤18	Ref			Ref		
>18	1.650	1.271–2.141	<0.001	1.434	1.096–1.875	0.009
Pathology			0.001			0.234
AC	Ref			Ref		
SQC	1.771	1.299–2.416	<0.001	1.357	0.927–1.989	0.117
Others	1.064	0.522–2.168	0.865	0.857	0.410–1.788	0.680
Differentiation			<0.001			<0.001
I	Ref			Ref		
II	1.465	0.923–2.324	0.105	1.248	0.784–1.988	0.350
III	2.718	1.715–4.310	<0.001	2.132	1.333–3.409	0.002
IV	2.570	1.178–5.611	0.018	2.054	0.928–4.546	0.076
ELN count			<0.001			<0.001
≤12	Ref			Ref		
>12	0.561	0.420–0.749	<0.001	0.586	0.437–0.786	<0.001

AC: adenocarcinoma; SQC: squamous cell carcinoma; Ref: reference; NS: *P* > 0.05 on univariate analysis.

## Discussion

Pathologic LN status is considered as one of the most important survival factors for EC. Patients with LN metastasis have a higher risk of disease recurrence and poorer prognosis. LN  sampling or dissection plays an important role in precise nodal staging and remains crucial for the appropriate delivery of adjuvant therapies. Increased extent of lymphadenectomy is associated with improved survival.^19^, ^20^, ^21^, ^22^, ^23^ Therefore, lymphadenectomy of some extent is required. However, what constitutes optimum lymphadenectomy to maximize survival, especially in pT1N0M0 EC is controversial.

Using data extracted from the United States SEER database, we demonstrated that ELN count in resected pT1N0M0 EC patients was an independent prognostic factor for both OS and ECSS. Although we were not able to ascertain a threshold of mortality benefit, we recommended more than 12 LNs to be examined. Patients who received induction chemo/radiotherapy were also included in this study, thus the findings can be generalized to those treated with preoperative therapy.

Our findings are compatible with results from previous studies.^19^, ^20^, ^24^ Rizk and colleagues^19^ performed a retrospective review using data from the Worldwide Esophageal Cancer Collaboration database. Greater extent of lymphadenectomy was associated with increased survival for patients with pT1N0M0 EC and optimum lymphadenectomy was 10 to 12 nodes. Liu *et al*.^20^ reviewed their experience of 666 patients who underwent esophagectomy and found that the number of resected LNs is an independent prognostic factor for the survival of node‐negative esophageal squamous cell carcinoma (ESCC) patients. The minimum resection number recommended for accurate staging is 16. Yu and colleagues^24^ retrospectively studied 194 pN0 ESCC patients who underwent radical esophagectomy. They reported that LN count exhibited prognostic significance in pN0 ESCC. In addition, the minimum number of LNs that should be removed in pN0 ESCC is probably 14.

However, a nationwide, population‐based cohort study indicated that more extensive LN clearance during surgery for EC may not improve survival. The study included 1044 EC patients who had undergone esophagectomy between 1987 and 2010 in Sweden, with follow‐up until 2012. Analyzed as a linear variable, a higher number of LNs removed did not influence the overall 5‐year mortality (adjusted HR = 1.00, 95% CI = 0.99 to 1.01).^25^ However, as an observational study, bias from confounding can never be excluded.

Although the mechanisms underlying the impact of ELN count on prognosis remain uncertain, there are several possible reasons for our finding that improved survival is related to higher ELN count in pT1N0M0 EC. Firstly, although we dissected enough LNs, the pathologists might not have examined all of the LNs from an en‐bloc resected specimen which would lead to stage migration. Thompson and colleagues^26^ identified 119 patients who had undergone surgical resection for EC between 1997 and 2007, and who were classified as node‐negative. Relevant paraffin blocks were identified, and three additional levels, each 250 μm apart, were cut of all LNs. They reported that one patient was found to have a metastasis (>2 ㎜), eight patients (6.7%) had micrometastasis, and 22 patients (18.5%) had isolated tumor cells. This indicated an effect of incomplete pathologic examination. Secondly, it is possible that a greater number of ELNs can be interpreted to be associated with lower risk of undiscovered positive lymph nodes, which meant that a better and more accurate staging. Node‐negative patients with a lower number of ELNs may in fact have had positive nodal involvement but were underdiagnosed because of inadequate LN staging, whereas node‐negative patients with more LNs examined were more likely to be truly free from nodal involvement.^23^ Thirdly, the more extensive lymph nodes dissection may reflect the adequacy of surgical, pathological and institutional care provided by the treatment team. A previous study identified that high‐volume hospitals had better late survival rates with EC resection than lower‐volume ones.^27^

The current study had several limitations. Firstly, it was a retrospective study and hence some inherent biases were inevitable. Secondly, there is no standardized protocol for removing and counting lymph nodes across institutions. Therefore, the ELN count may vary with pathologic processing, and counts in some institutions may include only LN fragments, while others rely on complete LNs^28^. Thirdly, detailed information on patients' comorbidity, performance status, pulmonary function, the length of the tumor, type of esophagectomy (Sweet, Ivor Lewis or McKeown, open or minimally invasive), margin status and recurrence rates could not be obtained from the SEER database. Lastly, the ELN count is recorded in the SEER database, but the location of each lymph node retrieval is not. Thus, determining the location of LNs resected or sampled was not possible.

In conclusion, the results of this study suggested that ELN count is independently associated with long‐time survival outcomes in resected pT1N0M0 EC patients. We recommend more than 12 LNs should be examined for accurate staging of operable pT1N0M0 EC. Because of the inherent limitations, further large‐scale cohort studies are needed to validate our findings and to explore the potential mechanisms underlying the prognostic implications of ELN count.

## Disclosure

The authors have no conflicts of interest to disclose.

## References

[1] Bray F , Ferlay J , Soerjomataram I , Siegel RL , Torre LA , Jemal A . Global cancer statistics 2018: GLOBOCAN estimates of incidence and mortality worldwide for 36 cancers in 185 countries. CA Cancer J Clin 2018; 68: 394–424. 30207593 10.3322/caac.21492

[2] Pennathur A , Gibson MK , Jobe BA , Luketich JD . Oesophageal carcinoma. Lancet 2013; 381: 400–12. 23374478 10.1016/S0140-6736(12)60643-6

[3] Muto M , Minashi K , Yano T *et al* Early detection of superficial squamous cell carcinoma in the head and neck region and esophagus by narrow band imaging: A multicenter randomized controlled trial. J Clin Oncol 2010; 28: 1566–72. 20177025 10.1200/JCO.2009.25.4680 PMC2849774

[4] Nagami Y , Tominaga K , Machida H *et al* Usefulness of non‐magnifying narrow‐band imaging in screening of early esophageal squamous cell carcinoma: A prospective comparative study using propensity score matching. Am J Gastroenterol 2014; 109: 845–54. 24751580 10.1038/ajg.2014.94 PMC4050526

[5] Rice TW , Ishwaran H , Ferguson MK , Blackstone EH , Goldstraw P . Cancer of the esophagus and esophagogastric junction: An eighth edition staging primer. J Thorac Oncol 2017; 12: 36–42. 27810391 10.1016/j.jtho.2016.10.016 PMC5591443

[6] Rice TW , Rusch VW , Ishwaran H , Blackstone EH , Worldwide Esophageal Cancer Collaboration . Cancer of the esophagus and esophagogastric junction: Data‐driven staging for the seventh edition of the American Joint Committee on Cancer/International Union Against Cancer Cancer Staging Manuals. Cancer 2010; 116: 3763–73. 20564099 10.1002/cncr.25146

[7] Wijnhoven BP , Tran KT , Esterman A *et al* An evaluation of prognostic factors and tumor staging of resected carcinoma of the esophagus. Ann Surg 2007; 245: 717–25. 17457164 10.1097/01.sla.0000251703.35919.02 PMC1877056

[8] Wani S , Drahos J , Cook MB *et al* Comparison of endoscopic therapies and surgical resection in patients with early esophageal cancer: A population‐based study. Gastrointest Endosc 2014; 79: 224–32.e1. 24060519 10.1016/j.gie.2013.08.002 PMC4042678

[9] Ancona E , Rampado S , Cassaro M *et al* Prediction of lymph node status in superficial esophageal carcinoma. Ann Surg Oncol 2008; 15: 3278–88. 18726651 10.1245/s10434-008-0065-1

[10] Pennathur A , Farkas A , Krasinskas AM *et al* Esophagectomy for T1 esophageal cancer: Outcomes in 100 patients and implications for endoscopic therapy. Ann Thorac Surg 2009; 87: 1048–54; discussion 1054–5. 19324126 10.1016/j.athoracsur.2008.12.060 PMC2912110

[11] Deng J , Liang H , Wang D *et al* Enhancement the prediction of postoperative survival in gastric cancer by combining the negative lymph node count with ratio between positive and examined lymph nodes. Ann Surg Oncol 2010; 17 (4): 1043–51. 20039218 10.1245/s10434-009-0863-0

[12] Osarogiagbon RU , Ogbata O , Yu X . Number of lymph nodes associated with maximal reduction of long‐term mortality risk in pathologic node‐negative non‐small cell lung cancer. Ann Thorac Surg 2014; 97 (2): 385–93. 24266949 10.1016/j.athoracsur.2013.09.058 PMC3946669

[13] Dolan RD , McSorley ST , Horgan PG , McMillan DC . Determinants of lymph node count and positivity in patients undergoing surgery for colon cancer. Medicine (Baltimore) 2018; 97: e0185. 29595652 10.1097/MD.0000000000010185 PMC5895435

[14] Malleo G , Maggino L , Ferrone CR *et al* Number of examined lymph nodes and nodal status assessment in distal pancreatectomy for body/tail ductal adenocarcinoma. Ann Surg 2018. 10.1097/SLA.0000000000002781 29672406

[15] O'Mahony M . Sensory Evaluation of Food: Statistical Methods and Procedures. Marcel Dekker, New York 1986.

[16] Greenwood PE , Nikulin MS . A Guide to Chisquared Testing. Wiley, New York 1996.

[17] Camp RL , Dolled‐Filhart M , Rimm DL . X‐tile: A new bio‐informatics tool for biomarker assessment and outcome‐based cut‐point optimization. Clin Cancer Res 2004; 10 (21): 7252–9. 15534099 10.1158/1078-0432.CCR-04-0713

[18] Cox DR . Regression models and life tables. J R Stat Soc B 1972; 34: 187–220.

[19] Rizk NP , Ishwaran H , Rice TW *et al* Optimum lymphadenectomy for esophageal cancer. Ann Surg 2010; 251: 46–50. 20032718 10.1097/SLA.0b013e3181b2f6ee

[20] Liu Q , Tan Z , Lin P *et al* Impact of the number of resected lymph nodes on postoperative survival of patients with node‐negative oesophageal squamous cell carcinoma. Eur J Cardiothorac Surg 2013; 44: 631–6. 23477926 10.1093/ejcts/ezt097

[21] Baba Y , Watanabe M , Shigaki H *et al* Negative lymph‐node count is associated with survival in patients with resected esophageal squamous cell carcinoma. Surgery 2013; 153: 234–41. 22980434 10.1016/j.surg.2012.08.001

[22] Hsu PK , Huang CS , Wang BY , Wu YC , Chou TY , Hsu WH . The prognostic value of the number of negative lymph nodes in esophageal cancer patients after transthoracic resection. Ann Thorac Surg 2013; 96: 995–1001. 23866797 10.1016/j.athoracsur.2013.04.098

[23] Zhu Z , Chen H , Yu W *et al* Number of negative lymph nodes is associated with survival in thoracic esophageal squamous cell carcinoma patients undergoing three‐field lymphadenectomy. Ann Surg Oncol 2014; 21: 2857–63. 24740827 10.1245/s10434-014-3665-y

[24] Yu Y , Wang W , Li Q *et al* Prognostic value of lymph node count on survival in pathologically node‐negative oesophageal squamous cell cancer. Interact Cardiovasc Thorac Surg 2018; 26: 407–12. 29177502 10.1093/icvts/ivx363

[25] van der Schaaf M , Johar A , Wijnhoven B , Lagergren P , Lagergren J . Extent of lymph node removal during esophageal cancer surgery and survival. J Natl Cancer Inst 2015; 107. 10.1093/jnci/djv043 25748792

[26] Thompson SK , Ruszkiewicz AR , Jamieson GG , Sullivan TR , Devitt PG . Isolated tumor cells in esophageal cancer: Implications for the surgeon and the pathologist. Ann Surg 2010; 252: 299–306. 20622664 10.1097/SLA.0b013e3181e61e15

[27] Birkmeyer JD , Sun Y , Wong SL , Stukel TA . Hospital volume and late survival after cancer surgery. Ann Surg 2007; 245: 777–83. 17457171 10.1097/01.sla.0000252402.33814.dd PMC1877074

[28] Gulack BC , Yang CF , Speicher PJ *et al* The impact of tumor size on the association of the extent of lymph node resection and survival in clinical stage I non‐small cell lung cancer. Lung Cancer 2015; 90 (3): 554–60. 26519122 10.1016/j.lungcan.2015.10.011 PMC4724282

